# How Are Gender Equality and Human Rights Interventions Included in Sexual and Reproductive Health Programmes and Policies: A Systematic Review of Existing Research Foci and Gaps

**DOI:** 10.1371/journal.pone.0167542

**Published:** 2016-12-21

**Authors:** Miriam Hartmann, Rajat Khosla, Suneeta Krishnan, Asha George, Sofia Gruskin, Avni Amin

**Affiliations:** 1 Women’s Global Health Imperative, RTI International, San Francisco, CA, United States of America; 2 World Health Organization, Geneva, Switzerland; 3 Research Triangle Institute Global India Private Limited, New Delhi, India; 4 School of Public Health, University of the Western Cape, Cape Town, South Africa; 5 Program on Global Health and Human Rights, University of Southern California, Los Angeles, CA, United States of America; University of North Carolina at Chapel Hill, UNITED STATES

## Abstract

The importance of promoting gender equality and human rights in sexual and reproductive health (SRH) programmes and policies has been affirmed in numerous international and regional agreements, most recently the 2030 Agenda for Sustainable Development. Given the critical role of research to determine what works, we aimed to identify research gaps as part of a broader priority setting exercise on integrating gender equality and human rights approaches in SRH programmes and policies. A systematic literature review of reviews was conducted to examine the question: what do we know about how research in the context of SRH programmes and policies has addressed gender equality and human rights and what are the current gaps in research. We searched three databases for reviews that addressed the research question, were published between 1994–2014, and met methodological standards for systematic reviews, qualitative meta-syntheses and other reviews of relevance to the research question. Additional grey literature was identified based on expert input. Articles were appraised by the primary author and examined by an expert panel. An abstraction and thematic analysis process was used to synthesize findings. Of the 3,073 abstracts identified, 56 articles were reviewed in full and 23 were included along with 10 from the grey literature. The majority focused on interventions addressing gender inequalities; very few reviews explicitly included human rights based interventions. Across both topics, weak study designs and use of intermediate outcome measures limited evidence quality. Further, there was limited evidence on interventions that addressed marginalized groups. Better quality studies, longer-term indicators, and measurement of unintended consequences are needed to better understand the impact of these types of interventions on SRH outcomes. Further efforts are needed to cover research on gender equality and human rights issues as they pertain to a broader set of SRH topics and populations.

## Introduction

Sexual and reproductive health (SRH) affects, and is affected by the intersectionality of people’s personal experiences and relationships and by the broader structural context of their lives which shapes their overall health and well-being. This context includes gender inequality as a determinant on its own and in combination with other social and economic inequalities including: unequal power dynamics in interpersonal relationships, harmful gender and other socio-cultural norms and practices, limited economic circumstances, lack of access to education, limited employment opportunities, poor living conditions, disability, ethnicity, as well as the challenging political and legal environments where they live[1]. Studies have shown that harmful gender norms that promote male dominance over women prevent women from practicing safer sex, limit their use of contraceptives, and increase their risk of STIs, including HIV[2]. Similarly, research has also shown a relationship between violation and neglect of human rights and negative health outcomes and emphasizes the need to better integrate human rights approaches into interventions, particularly with attention to provider training, service delivery, raising awareness and capacity building[3, 4].

In the past two decades, great strides have been made in the development of norms and standards related to gender equality and human rights pertaining to SRH and the interpretation and application of existing standards to different areas of SRH programming and policy making. This is true at the level of international policy, as well as at policy and programmatic levels within countries. The centrality of addressing interconnections between gender equality, human rights, and SRH, was recognized in the Cairo and Beijing agreements, the World Health Organization’s Reproductive Health Strategy[5], and most recently the 2030 Agenda for Sustainable Development and the accompanying Sustainable Development Goals (SDGs). Not only is the achievement of gender equality a goal in itself (i.e. Goal 5), but there is a specific target within that goal on universal access to SRH and rights (i.e. target 5.6), in addition to the target(i.e. target 3.7) on sexual and reproductive health in the health goal (i.e. Goal 3).[6]

The achievement of the highest attainable standard of SRH is thus closely linked with the extent to which gender equality and people’s human rights–such as the rights to non-discrimination, to privacy and confidentiality, to life, liberty, and security, as well as the rights to education, information and health–are respected, protected and fulfilled. In its recently adopted General Comment on the right to sexual and reproductive health, the UN Committee on Economic, Social and Cultural Rights, emphasizes that this includes, a “set of freedoms and entitlements”. The freedoms, according to the Committee, include the right to make free and responsible decisions and choices, free of violence, coercion and discrimination, over matters concerning one’s body and sexual and reproductive health. The entitlements, the Committee explains, include unhindered access to a whole range of health facilities, goods, services and information, which ensure all people full enjoyment of sexual and reproductive health.[6]

While progress has been made in understanding how some dimensions of gender inequalities and violations of human rights shape SRH outcomes, as well as in developing and evaluating interventions that promote gender equality in the context of some SRH programmes and policies, much remains to be done to address these issues systematically. Research has a critical role to play in effectively identifying and addressing gender inequalities and human rights by developing an understanding of what’s been done and what remains to be done. As a first step to determine what is known, a systematic literature review of reviews was conducted to address the following question: what do we know about how research in context of SRH programmes and policies has addressed gender equality and human rights and what are the current gaps in research? This review was conducted to inform a larger research priority setting exercise that is being undertaken to identify what research should be prioritized to strengthen the integration of effective gender equality interventions and human rights approaches in SRH programmes and policies. This paper summarizes the results of the review.

## Methods

The systematic literature review of reviews was guided by a protocol (See S1 Protocol) that identified the search strategy including search terms, inclusion and exclusion criteria, and a data abstraction process. The choice of conducting a review of reviews rather than primary intervention studies was guided by the necessity of including the broad scope of topics covered within the field of SRH.

### Search strategy

An automated search strategy was undertaken in 2014 to identify literature reviews, systematic reviews and meta-analyses. Three priority databases that typically index most systematic and other types of literature reviews were searched—Scopus, PubMed, and Cochrane—using a list of preliminary search terms related to gender equality, human rights, and SRH that included terms such as gender, gender norms, equality, equity, and sexual health. The preliminary search yielded a very large number of review articles). Therefore, some of the search terms were revised to minimize/remove duplicate terms (e.g., empowerment vs. women’s empowerment). Key words capturing important human rights concepts such as human rights, reproductive rights, and accountability were retained. Table 1, below, lists the initial terms used, as well as the revised terms.

**Table 1:** Search key words.

Topic	Search round 1 initial key words	Search round 1 revised key words	Search round 2 modifications
**Gender**	“gender” OR "gender equality" OR "gender inequality" OR "masculinity" OR "femininity" OR "gender norms" OR "power dynamics" OR "couple dynamics" OR "partner negotiation" OR "gender transformative" OR "gender responsive" OR "gender dynamics" OR "gender roles" OR "empowerment" OR "girl empowerment" OR "women empowerment" OR "social norms" OR "female subordination" OR "economic empowerment" OR "women’s roles" OR "reproductive roles" OR "gender sensitive" OR "women’s status" OR "gender integrated interventions" OR "gender sensitive" OR "women’s autonomy" OR "women’s choice" OR "women’s participation" OR "patriarchy" OR "sexual orientation" OR "transgender" OR "LGBT" OR "gender identity" OR "SOGI" OR "women’s decision-making" OR "couples decision-making"	"gender equality" OR "gender inequality" OR "masculinity" OR "femininity" OR "gender norms" OR "power dynamics" OR "partner negotiation" OR "gender transformative" OR "gender responsive" OR "gender dynamics" OR "gender roles" OR "empowerment" OR "women’s roles" OR "reproductive roles" OR "gender sensitive" OR "gender integrated interventions" OR "gender sensitive" OR "women’s autonomy" OR "women’s choice" OR "patriarchy" OR "sexual orientation" OR "transgender" OR "LGBT" OR "gender identity" OR "SOGI"	[Remained the same as revised key words]
**Human rights**	"equality" OR "equity" OR "stigma" OR "non-discrimination" OR "accountability" OR "privacy and confidentiality" OR "informed decision making" OR "participation" OR "availability" OR "accessibility" OR "acceptability" OR "quality of care" OR "sexual rights" OR "reproductive rights" OR "sexual and reproductive health rights" OR "women’s rights" OR "LGBT rights" OR "intersex rights" OR "respect" OR "disrespect"	"human rights" OR "reproductive rights" OR "sexual rights" OR "health rights" OR "women's rights" OR "LGBT rights" OR "intersex rights" OR "equity" OR "equality" OR "non-discrimination" OR “stigma” OR "accountability” OR “participation” OR “privacy and confidentiality”	[Remained the same as revised key words]
**SRH**	"sexual health" OR "reproductive health" OR “sexual and reproductive health” OR "reproductive choice" OR "contraceptive choice" OR "family planning" OR "gender based violence" OR "contraception" OR "STI" OR "HIV" OR "abortion" OR "sex education" OR "cervical cancer" OR "ovarian cancer" OR "breast cancer" OR "infertility" OR "violence against women" OR "maternal health" OR "maternal mortality" OR "maternal morbidity" OR "prolapse" OR "fistula" OR "FGM/C" OR "child marriage" OR "early marriage" OR "forced marriage" OR "menstruation" OR "menstrual hygiene"	"sexual health" OR "reproductive health" OR "reproductive choice" OR "contraceptive choice" OR "family planning" OR "contraception" OR "sex education"	“HIV/AIDS” OR “sexually transmitted infections” OR “maternal health” OR “ASRH” OR “infertility” OR “cervical cancer” OR “abortion” [These terms were chosen to reflect topics areas that were thought to be areas of greater research focus and thus topics that may yield further reviews, and were not topics currently being covered in other WHO research prioritization exercises (e.g. violence against women, female genital mutilation, and child marriage).]
**General (1)**	“laws” OR “policies” OR “systems”	[Removed given that articles on laws, policies, or systems were not excluded with other search terms]	N/A
**General (2)**	“systematic reviews” OR “reviews” OR “literature reviews” OR “meta-analysis” OR “scoping reviews” OR “narrative reviews” OR “qualitative reviews”	[Removed–publication type “review” or its equivalent used instead]	N/A

Finally, in addition to database searches, we consulted participants of an expert meeting that was convened in January 2015 to identify additional reviews from the grey literature that may have been missed in the databases[7].

### Inclusion and exclusion criteria

Articles, both peer reviewed and from the grey literature, that met the following criteria were included: (i) addressed the primary research question: what do we know about how research in context of SRH programmes and policies has addressed gender equality and human rights and what are the current gaps in research; (ii) published between 1994 and 2014 –the period between when ICPD raised global attention to the role of gender equality and human rights in SRH and when the search was conducted; and (iii) classified as reviews and met methodological standards (i.e. followed PRISMA guidelines) for systematic reviews, or qualitative meta-syntheses, or other reviews of relevance to the research question. For the purpose of this review, interventions were defined as actions seeking to promote change in a directed manner. For example, a human rights intervention may be an intervention aimed at empowering women and girls to know and claim their rights in context of sexual and reproductive health. Articles were selected if the role of gender or human rights was explicitly stated as the focus of an intervention or if a gender equality or human rights component was explicitly included within a broader SRH intervention. SRH outcomes of interest were limited to those included within the WHO Reproductive Health (RH) strategy. These include family planning (FP), unsafe abortion, sexually transmitted infections (STI) HIV, reproductive tract infections (RTIs), gender-based violence (GBV), menstrual conditions, urinary and fecal incontinence due to obstetric fistula, uterine prolapse, pregnancy loss, sexual dysfunction, female genital mutilation (FGM), antenatal, perinatal, postpartum and newborn care, cervical cancer, and infertility.[5]

Articles that were considered secondary analyses, discussions of literature, editorial discussions, or which did not provide sufficient detail regarding their review methodology were excluded. Articles were also excluded if: (i) they were published in a language other than English or Spanish (the languages accessible by the primary authors), or (ii) human rights, gender equality, or SRH outcomes were not explicitly discussed.

### Title, abstract, and article screening

Search results from each of the three priority databases were exported into Excel and titles and abstracts were reviewed by the primary author (MH) to identify articles that merited full text review. Articles were delineated into those that met review criteria based on the abstract (Y), may meet review criteria (M), or did not meet review criteria (N). Those articles identified as meeting (Y) or may meet (M) the review criteria were reviewed in full text. Findings were reviewed with other authors (SK, AA, RK) and decisions discussed.

### Study appraisal and synthesis

Selected reviews that still met the inclusion criteria, after full text evaluation, were abstracted for the following information: authors, year, title, journal, type of review, population focus, geographic focus, gender/human rights interventions covered, SRH topics covered, outcomes assessed, and research gaps/limitations. Reviews identified in the grey literature by the expert meeting participants in January 2015 were subjected to the same abstraction process.

## Results

### Literature Review and Search Process

**Fig 1:**
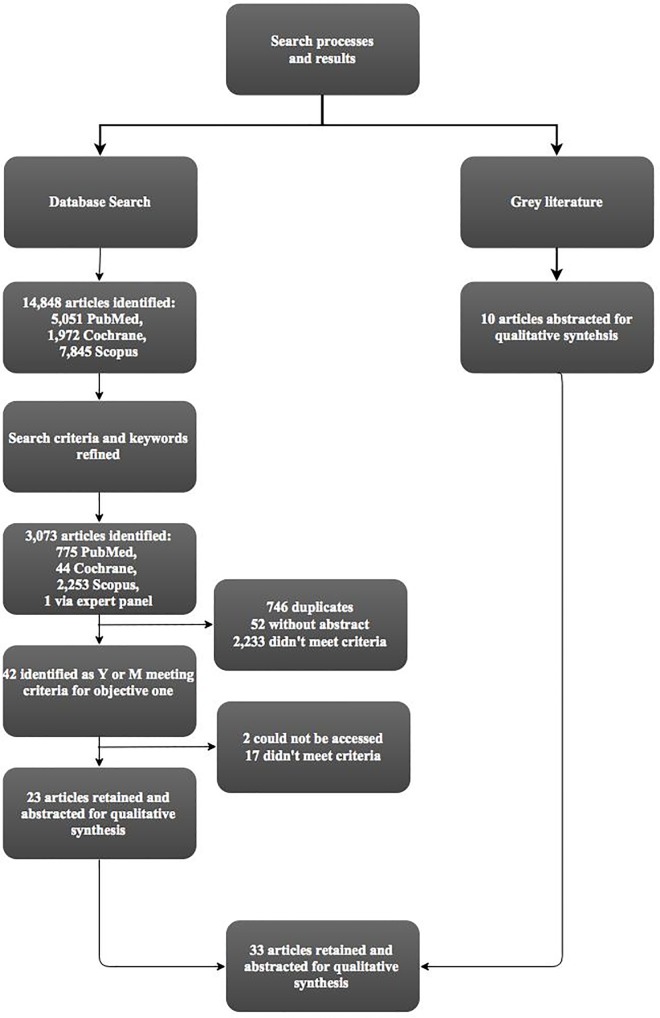
Search and screening process and results.

In total, we reviewed 3,073 abstracts from our peer-review literature search. We shortlisted 42 abstracts as either meeting (Y) or possibly meeting (M) the inclusion criteria for the review. These articles were retrieved for a full text review. Following a full text review 19 of the 42 articles were excluded as they did not meet the inclusion criteria. The 23 remaining articles were abstracted as per the process outlined above. In addition, ten reviews from the grey literature were reviewed and abstracted. Fig 1, below, outlines the search and screening process and reasons for article exclusion.

### Characteristics of Included Reviews

Ten out of the thirty-three publications (i.e. 23 peer reviewed articles and 10 reports from grey literature) were classified as systematic reviews, all the peer-reviewed articles were published in health-related journals, and the majority of articles either took a global perspective or focused on low and middle-income countries. Despite the wide time range specified for inclusion (1994–2014), the oldest gender focused review selected was published in 2003 and the oldest human rights focused review article was published in 2011. More specific characteristics of the included review articles are presented below and outlined in tables that are available as supporting information to this manuscript (S1 and S2 Tables).

### What’s the state of the research?

Based on the findings of the review the following section identifies research foci and gaps in order to inform research priority setting in relation to developing effective interventions and approaches to address gender equality and human rights within sexual and reproductive health programmes and policies. Results are broken down by key themes, such as research topic, location, target population, outcomes, and other methodological issues.

#### Research Topics

Human Rights: Only five review articles specifically assessed the impact of human rights interventions on SRH outcomes. These included interventions targeted at the health facility[8–10], community[10, 11], and policy[12] levels. Given this dearth of information, the primary topical gap is a lack of explicit focus on human rights interventions in SRH research. Furthermore, the reviews that did exist tended to focus on one or two human rights principles (e.g. participation, empowerment) as against a comprehensive inclusion of human rights interventions in relation to SRH. Furthermore, the review highlights the need to look beyond traditional ‘outcomes’ and to focus more on processes and staggered effects over time. [8]

Gender equality: The majority of articles (N = 29) represented reviews of gender-equality focused interventions. These reviews most frequently assessed impact of gender equality or human rights on HIV outcomes (N = 18), followed by other SRH outcomes such as FP (N = 7), gender-based violence (N = 8), and maternal health (N = 6). The gender equality interventions focused on male involvement, women’s empowerment, addressing gender roles—such as those relating to communication and decision-making, and biomedical interventions addressing gender-based barriers to SRH (e.g. female-controlled HIV prevention methods). Several reviews examined whether strategies reinforced (i.e. perpetuated unequal gender relations), accommodated (i.e. took into account men and women’s unequal roles and power but did not change them) or were transformative (i.e. attempted to change unequal gender power relations and norms)[13–15]. Reviews also indicated that gender-equality interventions in this area have ranged from those that focused on structural factors such as community gender norms to others that focused on interpersonal gender power relations reflected in couple’s communication.

Intersection of Gender and Human Rights: While it is assumed that implicitly, interventions that address gender inequalities de facto are addressing some human rights concerns and vice versa, few reviews explicitly addressed both gender inequalities and human rights. One of the five human rights focused reviews examined interventions specifically designed to promote FP demand and access using rights-based approaches, and also included an intervention to address inequitable gender norms[10]. Similarly, three reviews of gender-equality focused interventions were explicitly stated to be addressing human rights by framing interventions to address gender-based violence (GBV) or gender-barriers to FP as human rights issues[16–18].

SRH: Across both gender equality and human rights reviews, a comparison between the SRH topics addressed in the reviews and those included in the WHO RH strategy identified several gaps. The primary SRH topics covered by reviews of human rights interventions have been GBV and FP. Gender equality interventions included in the reviews have looked at a broader range of SRH topics (e.g., maternal health, FP/contraception, HIV/STIs). However, even within maternal health and FP, specific topics such as safe motherhood, healthy timing and spacing of pregnancy, and neonatal, child health and nutrition have received less attention. Other topics such as menstrual conditions, urinary and fecal incontinence due to obstetric fistula, uterine prolapse, infertility, and cervical cancer were relatively limited in their inclusion in the reviews. Similarly abortion and FGM, both topics that have clear gender equality and human rights associations, were rarely covered.

Laws and Policies: The reviews did not include many studies or interventions that examined the potential positive impacts of laws and policies promoting gender equality or human rights on SRH. One review focused on the negative impact of discriminatory laws around sex work, including their impact on voluntariness of medical care and access to health services; yet the review itself found limited data regarding the positive impact of changes in policies on SRH outcomes (e.g. how law influences sex workers ability to avoid STIs or to access SRH services as opposed to measuring levels of HIV infection)[12]. Another included some review of how changes in policies positively impact human rights related outcomes such as access to FP, participation of diverse stakeholders, or equity, nondiscrimination, and quality of SRH care. This latter review found that most policy level intervention studies were not true evaluations, but rather documentation of FP policy implementation and subsequent changes in FP access and uptake. It also found that while policies exist to support FP access and uptake, a human rights perspective to doing so is often not included. Some identification of factors necessary to ensure the fulfillment of individuals’ rights to FP, defined as availability, accessibility, acceptability and quality of contraceptives, and participation in decision-making, were found in this literature. However a need to better understand the association between human rights and SRH was acknowledged in many of the reviews, as was the need for better tools to monitor both health and human rights concerns.[10, 19] This was particularly noted for topic areas that are difficult to measure (e.g. abortion)[20].

#### Geographical Focus

Although several reviews attempted to include data on either global level or low- and middle-income country interventions, they found gaps in the geographical focus of the research studies they identified. For instance, within low- and middle-income countries there was a dearth of evidence from the Middle East and North Africa with more research occurring in sub-Saharan Africa[21]. Humanitarian or conflict-affected countries were often cited as missing from the evidence-base as well, particularly in relation to violence against women, an issue that can be exacerbated in these settings[17, 22]. When interventions have been conducted in these settings, the reviews noted they are often not rigorously evaluated and thus their effectiveness is unclear[17]. Finally, one review found that within South Asia, the majority of gender equality interventions across several SRH topics (i.e. RMNCH, HIV, GBV, and universal health care) took place in India[23].

#### Target Populations

The ways in which population of focus were defined in gender equality and human rights reviews differed. Gender equality reviews often included generic categories of women and/or men whereas reviews of human rights interventions tended to focus more on people who were marginalized such as female sex workers or women living with HIV. For example, two reviews of human rights interventions focused on sex workers, one of which reviewed policy and advocacy interventions to promote female sex workers’ rights and the other reviewed community empowerment initiatives designed to overcome barriers to female sex workers’ health and human rights[11, 12]. A third review focused on the pregnancy-related rights of HIV-positive women[9]. The final two, which focused on women and children and women and men broadly speaking–reviewed elements of healthcare according to human rights principles such as availability, accessibility, acceptability, and quality of care[8, 10]. Despite the more clear focus on marginalized populations within human rights reviews, reviews with a focus on lesbian, gay, bi-sexual, or transgender individuals were not found suggesting both a heteronormative lens to the existing research base and the invisibility of gender expressions that differ from the cultural norm. Additionally, while four reviews addressed adolescents, two of which focused exclusively on this population (one on African American youth and another on Latino youth in the US and Mexico)[14, 15, 17, 24], concentration on this population, which often faces stigma and discrimination in SRH service provision, was still relatively rare.

#### Outcomes

Gender, Human Rights, and SRH outcomes: Reflective of the range of topics and foci addressed across the reviews, outcome measures ranged accordingly. Gender related measures often included gender norms, partner communication, male involvement levels, decision-making or power dynamics, but also measurement of harmful outcomes, such as incidence of FGM, violence, or early marriage. The use of longer term outcomes like female life expectancy were rare, but were used on occasion, such as in one review examining outcomes of gender equitable policies[19]. While harmful SRH outcomes that were the intended focus of interventions were at times included, such as incidence of FGM, it was not common for interventions to measure unintended harm resulting from intervention efforts[22, 25, 26]. Furthermore, some reviews noted that male engagement approaches, in particular, suffer from greater inconsistences in measurement and in impact on SRH behaviours and health outcomes and thus, in understanding how male engagement might help or possibly hinder women’s empowerment.[21, 27, 28]

The framing of human rights measures similarly varied according to intervention goals. These ranged from exposure to empowerment interventions, to access to care, to longer term impacts, such as decriminalization of sex work. Accessibility and quality of services were commonly measured in areas such as FP to sex work[8–10, 12], whereas measures of stigma or voluntariness were used less consistently[9, 12]. This was the case even when the review included studies that explicitly included interventions promoting voluntary rights-based FP[10].

Across the reviews, the majority of SRH outcomes were related to changes in knowledge, attitude, or behaviors, and not clinical outcomes (e.g. STI or HIV prevalence). Related to this, self-report was commonly used to measure outcomes such as STI symptoms or male perpetration of VAW highlighting the methodological limitations of studies in the reviews.[22]

Cost, replicability, and scale: A majority of the studies or interventions covered in the reviews point to research on new innovations or small-scale interventions addressing gender equality in single settings. Only one review focused on examining the implementation of an intervention found to be effective in one setting, ‘Stepping Stones’, across multiple settings[29]. Therefore, more research is needed on replicability of pilot gender equality (and human rights) interventions across different settings and their scale up. In addition, only two of the reviews provided information on the costs, cost-effectiveness, and sustainability of gender equality and human rights-based interventions in SRH programmes and policies[15, 30]. One review, which focused exclusively on cost and cost-effectiveness, found as well that most cost-related data comes from pilot and single-site studies, again pointing towards a research gap around intervention scale[30].

#### Other Methodological Gaps

A number of methodological limitations of existing research on gender equality and human rights-based interventions and SRH emerged. The majority of identified reviews included studies using either qualitative or quantitative methods but not both, and none of the reviews undertook a qualitative meta-ethnographic or meta-synthesis/analysis process. Methodological rigor may also vary according to intervention approach and region of implementation. One review, which examined research design according to gender accommodating versus transformative interventions, found that accommodating interventions less commonly used a mixed methods approach and those attempting to transform gender norms tended to use qualitative only designs. The review also identified less use of randomized controlled trials in South Asia as compared to other regions of the world.[23] Reviews also identified studies with poor quality design highlighting the need for better quality research studies that not only rely on randomized controlled trials, but also case series, cohort studies and use qualitative research methodologies that are well recognized in order to instill reasonable confidence in the findings. Diversity in outcome measures (as reported in the section on outcomes) highlight the need for a validated and consistent range of outcome measures of gender equality and human rights interventions[11, 21, 22, 25–27, 31–36]. Consideration for a longer timeframe of measurement in order to assess whether positive outcomes are sustained over time was often highlighted[15, 17, 21, 23, 25, 26, 29, 33, 34]. The reviews of gender equality interventions (e.g. social norm change) tended to have limited time frames making it difficult to measure behavior change or the impact on health outcomes.

## Discussion

Nearly 20 years after ICPD Programme of Action (1994) and the Beijing Platform for Action (1995) highlighted the importance of addressing gender equality and human rights in health and specifically SRH programmes, there has been an accumulation of interventions, programming and evaluations that have enabled researchers to review bodies of evidence. This review of reviews provides an overview of that body of evidence, highlighting what the focus and subsequent gaps have been across topics, population and geographic focus, and methodological approaches.

While interventions addressing gender equality and human rights were recognized as important more than a decade ago, our review found that research on gender equality has received more attention than human rights. This is reflected in the relative paucity of peer reviewed reviews on human rights, particularly in the public health literature. A myriad of factors, including limited funding and limited understanding of human rights, may contribute to this finding. As a result, this body of evidence suffers from additional challenges including selective or ad hoc application of some elements (e.g. non-discrimination, participation) of a human rights based approach as against a comprehensive application; data considerations; and lastly implicit integration of what are called human rights considerations, instead of explicit and comprehensive integration of human rights as internationally understood which impairs study design and evaluations.[1, 8]

Although the reviews on gender equality provide a much wider body of evidence than human rights, it’s still challenged by gaps that impede our ability to understand the effectiveness of these interventions. Not surprisingly, given women’s often socially prescribed lower status in communities worldwide, the interventions included in the reviews primarily focused on women and women’s SRH outcomes. While interventions have clearly targeted gender equality for women from multiple levels ranging from the interpersonal to the structural, understanding and measuring change from both women and men’s perspectives is still inadequately done. Specifically, in line with recent ethical and safety recommendations on VAW intervention research[37], evaluations of interventions should measure potential harms or unintended consequences, as well as outcomes in terms of the individual and in relation to social norms. Moreover, there is a need to better understand whether and what types of interventions lead to resistance to prevailing inequitable gender norms among men and how resistance to norms impacts men for the sake of their own SRH (as partners, fathers, and community members). The latter is of particular importance given both, the challenge of engaging men in these issues and the need to engage them to address their contribution to maintaining existing gender inequalities that inhibit women’s health. While much remains to be learned on effective male engagement, motivating men based on the contributions they can make, as well as their own benefits has been highlighted as an important approach and as such, reliable and valid measurement of these outcomes needs to be incorporated.[21, 28, 32]

Finally, several gaps identified by this review, including the short timeframes of research studies, the limited geographical spread of research, the minimal inclusion of a wider variety of populations, and the missing focus on policy research and on cost, replicability and scale of interventions hinder the field’s ability to progress in integrating gender equality and equal human rights in SRH programmes and policies. For example, shorter timeframes for measuring research outcomes inhibit our ability to understand longer term change–a challenge of great importance for issues such as these, where interventions must support intensive changes in social norms and sustain changes in SRH behaviours. Without longer term research frameworks and funding support to measure and evaluate impact of gender equality and human rights interventions in SRH programmes and policies, we will continue to rely on shorter term outcome measures that can only capture shifts in more proximate knowledge and attitudes, rather than truly understanding what is required to change social, cultural and other norms and behaviours. As another example, the limited attention to replicability of interventions included in the reviews prevent us from understanding how different socio-cultural and economic context would affect whether or not a particular gender equality intervention or human rights approach would work across settings. Similarly, the pilot nature of interventions or studies included in the reviews makes it difficult to know and understand whether these would work or not if implemented on a larger scale. Funding agencies have an important role to play in shaping where, how, and for how long research projects occur in order to allow for measuring longer term, community-wide and larger scale impacts across different settings

This review of reviews has several limitations. One limitation was the search focused on databases that primarily capture the public health literature. Although we did identify articles from disciplines other than public health (e.g. legal, policy), all articles meeting review criteria ultimately came from public health journals. This may have somewhat limited the scope of which gender and human rights interventions were considered. However, given the need to identify articles measuring SRH outcomes, we expect that this limitation should be minimized. Another limitation was the restriction of inclusion criteria of reviews published in English and Spanish. This was due to the language capabilities of the authors; yet, given that the inclusion of Spanish reviews identified no additional reviews meeting criteria, it’s likely that the addition of other languages would have yielded similarly low returns. Additionally, although a large number of potential search terms (key words) were initially identified, we were compelled to revise the search strategy due to the volume of results. This, in addition to our focus on review of reviews, contributed to the varied nature of interventions captured under the broad categories of gender equality and human rights and as such, our inability to make nuanced observations about the research gaps within specific gender equality or human rights intervention approaches. For example, among human rights interventions, we were unable to review interventions at the individual, community, health care, or national level or across identified principles such as non-discrimination, availability, accessibility, acceptability, quality of care, decision-making, or participation[4]. Without explicit search terms reflecting the many levels of intervention, we cannot be certain that the exclusion of certain topics is reflective of an actual lack of research across these many areas. On the other hand, the broad search terms used should have captured research framed as gender equality or human rights, thus pointing again to a lack of conceptualization of principles of non-discrimination, availability, participation etc., as part of a human rights framework.

## Conclusion

This review highlights that progress has been made over the last fifteen years on research related to inclusion of gender equality and human rights interventions in SRH policies and programming, but fundamental gaps remain. Much more has been published on gender equality interventions than human rights-based interventions in the SRH literature. Research relating to both gender equality and human rights based interventions has been published on topics such as HIV/AIDS, SRH, more broadly, and to a limited extent on FP. There is a need to strengthen research methods and measurement of outcomes to capture long-term sustained changes in SRH behaviours and biological health outcomes[21, 22]. Attention to human rights in intervention research although limited and ad hoc in nature is increasing, although here too publication has been greatest in relation to HIV and FP. Further investment, documentation, research and development of methodologies is needed in order to capture a) the pathways by which gender equality and human rights interventions can improve SRH outcomes; and b) how we address intersecting forms of inequalities and discrimination along with gender and human rights concerns faced by specific sub-populations and marginalized groups in relation to their SRH. Increased coordination between gender and human rights focused actors could improve their ability to more holistically incorporate human rights and gender into intervention design, implementation, and evaluation thereby strengthening the evidence base.

## Supporting Information

S1 Protocol (DOCX)

S1 Table Gender-focused reviews. (DOCX)

S2 Table Human rights focused reviews. (DOCX)
