# Supplementary material for: How Are Gender Equality and Human Rights Interventions Included in Sexual and Reproductive Health Programmes and Policies: A Systematic Review of Existing Research Foci and Gaps
Source: PLoS One. 2016 Dec 21;11(12):e0167542. doi: 10.1371/journal.pone.0167542 (PMC5176262; doi:10.1371/journal.pone.0167542)
Supplement: S1 Protocol — (DOCX) [file pone.0167542.s001.docx]

# WHO Systematic Review of Reviews Protocol: Promoting Gender and Human Rights in SRH

## Objective

The objective of this review will be to map existing reviews to understand the nature of current evidence and research gaps regarding the promotion of gender equality and human rights in sexual and reproductive health (SRH) interventions^[[1]](#footnote-1)^. This will serve as an input to the broader exercise of setting research priorities in these domains.

## Background

Sexual and reproductive health effects, and is affected by, people’s personal experiences and relationships and by the broader context of their lives, including the gendered context, which shapes their economic circumstances, education, employment opportunities, living conditions, family structures and cultural, political and legal environments. Recognizing these interconnections, the Cairo and Beijing agreements, the MDGs and the WHO Global Reproductive Health Strategy propose a multilevel and multisectoral approach to improving all aspects of people’s lives, including but not limited to their sexual and reproductive health and related human rights.

Much has been achieved in the last twenty years since ICPD and Beijing at the level of international policy dialogue and at policy and programmatic levels within countries. Concepts have been clarified; indicators selected; data compiled; programs designed and evaluated; technologies expanded and improved; best practices identified and adopted; and international and government agencies and non-governmental organizations (NGOs) activated. However, despite such progress, recent systematic reviews highlight various gaps in research (Futures Group, 2014; Kraft, 2014). In particular, knowledge gaps remain on how to effectively promote gender equality and human rights within SRH interventions, as well as how these attempts can improve SRH outcomes.

To address this gap the overall project aims to identify thematic and cross-cutting research priorities for integrating gender equality and human rights in SRH health policies and programs through the identification of research gaps and priorities in this field. Specifically this protocol will contribute to the aims of the WHO’s overall project by conducting a review of two priority areas:

1. existing evidence and gaps on interventions to address/promote gender equality in the context of SRH programs and policies; and
2. existing evidence and gaps on interventions to address/promote human rights in the context of SRH programs and policies.

These gaps will be the basis for developing domains and research questions at a meeting of experts in January 2015. This will lead to the process of research prioritisation after a meeting in January.

## Search Strategy

To conduct a review of literature reviews, we will primarily use an automated search strategy, which will be supplemented by the identification of additional references through the panel of experts identified to participate in this review.

### Time period

Our search will be limited to review articles, published between 1994 and 2014. The selection of 1994 as the start-date of the review is based on the consensus that emerged from the International Conference on Population and Development in Cairo, which broadened the focus of population policies to an approach which more broadly promoted sexual and reproductive health, and served as the background to the WHO Reproductive Health Strategy.

### Geographic focus

Given the fact that much of the existing evidence may be drawn from high income countries, the search will not be limited by a select geographic region. Rather, we will denote whether identified articles represent evidence from high or middle/low income countries (as defined by the World Bank) during our data extraction process.

### Language

The search will initially be limited to only those articles published in English or Spanish. Depending on the number and relevance of articles identified, the search may be expanded to other languages. This decision will be made by WHO in consultation with the informal advisory group of experts.

### Databases

The following databases will be searched for both qualitative and quantitative literature reviews, systematic reviews, and meta-analysis that have been published in either peer-reviewed and/or grey literature:

- [Scopus](http://www.scopus.com/)
- [Cochrane](http://onlinelibrary.wiley.com/cochranelibrary/search/)
- [Pubmed](http://www.ncbi.nlm.nih.gov/pubmed/advanced)

### Search terms

Numerous search terms will be used representing the fields of gender, human rights and SRH. The following table provides a list of the key search terms to be used by category. Searches will be conducted using the following combination of search terms:

- [1] or [2] and [3]

Search terms will be revised based on inputs by the selected group of advisers. Each revision of the terms will be clearly documented in this protocol.

| Topic | Initial key words | Revised key words |
| --- | --- | --- |
| [1] Gender | “gender” OR "gender equality" OR "gender inequality" OR "masculinity" OR "femininity" OR "gender norms" OR "power dynamics" OR "couple dynamics" OR "partner negotiation" OR "gender transformative" OR "gender responsive" OR "gender dynamics" OR "gender roles" OR "empowerment" OR "girl empowerment" OR "women empowerment" OR "social norms" OR "female subordination" OR "economic empowerment" OR "women’s roles" OR "reproductive roles" OR "gender sensitive" OR "women’s status" OR "gender integrated interventions" OR "gender sensitive" OR "women’s autonomy" OR "women’s choice" OR "women’s participation" OR "patriarchy" OR "sexual orientation" OR "transgender" OR "LGBT" OR "gender identity" OR "SOGI" OR "women’s decision-making" OR "couples decision-making" | "gender equality" OR "gender inequality" OR "masculinity" OR "femininity" OR "gender norms" OR "power dynamics" OR "partner negotiation" OR "gender transformative" OR "gender responsive" OR "gender dynamics" OR "gender roles" OR "empowerment" OR "women’s roles" OR "reproductive roles" OR "gender sensitive" OR "gender integrated interventions" OR "gender sensitive" OR "women’s autonomy" OR "women’s choice" OR "patriarchy" OR "sexual orientation" OR "transgender" OR "LGBT" OR "gender identity" OR "SOGI" |
| [2] Human rights | "equality" OR "equity" OR "stigma" OR "non-discrimination" OR "accountability" OR "privacy and confidentiality" OR "informed decision making" OR "participation" OR "availability" OR "accessibility" OR "acceptability" OR "quality of care" OR "sexual rights" OR "reproductive rights" OR "sexual and reproductive health rights" OR "women’s rights" OR "LGBT rights" OR "intersex rights" OR "respect" OR "disrespect" | "human rights" OR "reproductive rights" OR "sexual rights" OR "health rights" OR "women's rights" OR "LGBT rights" OR "intersex rights" OR "equity" OR "equality" OR "non-discrimination" OR “stigma” OR "accountability” OR “participation” OR “privacy and confidentiality” |
| [3] SRH | "sexual health" OR "reproductive health" OR “sexual and reproductive health” OR "reproductive choice" OR "contraceptive choice" OR "family planning" OR "gender based violence" OR "contraception" OR "STI" OR "HIV" OR "abortion" OR "sex education" OR "cervical cancer" OR "ovarian cancer" OR "breast cancer" OR "infertility" OR "violence against women" OR "maternal health" OR "maternal mortality" OR "maternal morbidity" OR "prolapse" OR "fistula" OR "FGM/C" OR "child marriage" OR "early marriage" OR "forced marriage" OR "menstruation" OR "menstrual hygiene" | "sexual health" OR "reproductive health" OR "reproductive choice" OR "contraceptive choice" OR "family planning" OR "contraception" OR "sex education" OR “HIV/AIDS” OR “sexually transmitted infections” OR “maternal health” OR “ASRH” OR “infertility” OR “cervical cancer” OR “abortion”^[[2]](#footnote-2)^ |

In addition to the above terms, the publication type field offered in various databases as a search criteria will be selected as “review” or whatever alternative term is available in the database.

## Review Selection

The results of each search will be exported into Excel along with details on the search terms used database searched and date of search. An initial review of the article title and if needed abstract will be completed by the primary reviewer and articles will be marked as either ‘yes’ ‘no’ or ‘maybe’ for inclusion using the selection criteria outlined below. The full text of those articles identified as ‘yes’ or ‘maybe’ will be reviewed to confirm that they meet the eligibility criteria. For those articles identified as not relevant for inclusion the reason for non-inclusion will be recorded in a separate column on the spreadsheet.

### Selection criteria

| Inclusion | Exclusion |
| --- | --- |
| - - Published between 1994 and 2014.   - Systematic reviews: structured search of bibliographic and other databases to identify relevant literature; use of transparent methodological criteria to exclude papers not meeting an explicit methodological benchmark; presentation of rigorous conclusions about outcomes.   - Narrative reviews: purposive systematic sampling of the literature of interest; use of theoretical or topical criteria to include papers on the basis of type relevance and perceived significance with the aim of summarizing discussing and critiquing conclusions.   - Qualitative meta-syntheses or meta-ethnographies: structured search of bibliographic and other databases to identify relevant literature; use of transparent methods to draw together theoretical products with the aim of elaborating and extending theory.   - Reviews which answer what gender or human rights based interventions can be used to promote SRH   - Reviews which answer how gender and human rights based interventions have affected SRH outcomes   - Sufficient detail on the interventions reviewed is provided such that the review’s suitability for inclusion can be assessed   - Published in English or Spanish   - Reviews that:     - explicitly frame themselves as addressing gender or human rights (i.e. exclude other interventions, which may aim to increase SRH access or quality of care if their intention to address human rights is not clearly articulated ). An explicit reference to human rights should include the terms ‘human rights’, ‘reproductive rights’ or ‘women’s rights.’ An explicit reference to gender may include the terms ‘gender’, ‘empowerment’     - or are SRH interventions that explicitly include a gender or human rights component to the intervention and/or explicitly report on gender or human rights outcome, as defined in the point above. | - Secondary analyses (including qualitative meta-syntheses or meta-ethnographies) of existing data sets for the purpose of presenting cumulative outcomes from personal research programs. - Secondary analyses (including qualitative meta-syntheses or meta-ethnographies) of existing data sets for the purpose of presenting integrative outcomes from different research programs. - Discussions of literature included in contributions to theory building or critique. - Summaries of the literature for the purpose of information or commentary. - Editorial discussions that argued the case for a field of research or a course of action. - Papers whose abstract identified them as reviews but that lacked supporting evidence in the main text (e.g. details on the databases searched or the selection criteria). - Reviews which do not include gender, human rights and SRH outcomes of interest^[[3]](#footnote-3)^ as defined by the key search terms. |

## Data extraction

Additional data from those articles identified for inclusion will be abstracted using an abstraction table created in Excel. The table will be developed through discussions with the panel of advisors and will first be piloted by the primary reviewer by abstracting two sample articles. After collective review and discussion the table will be further refined and a common consensus on how to abstract the remaining articles will be agreed upon. The remaining articles will then be abstracted by the primary reviewer the results of which will be discussed by the entire panel of advisors. The following table represents the suggested information for extraction:

| Authors | Year | Title | Journal | Type of review | Geographic focus | Gender and human rights interventions covered | SRH topics covered | Outcomes assessed | Population focus | Research Gaps / Limitations |
| --- | --- | --- | --- | --- | --- | --- | --- | --- | --- | --- |
|  |  |  |  |  |  |  |  |  |  |  |

## Data synthesis

Once the final selection of articles has been agreed upon a qualitative content analysis of the articles will be conducted to identify what domains and research topics have already been addressed and what gaps exist. To present the results an analytical framework will be developed to categorize information from the reviews examined. This framework which will aim to categorize results by gender/rights domains will be further developed through discussion of abstraction results among the panel of advisors. If feasible and/or deemed necessary to provide a more comprehensive picture of the landscape results may also be categorized by SRH topic. However, if the latter is done, the review may start with a limited number of SRH topics, pointing to the need for additional funding/time to analyse the remaining topics. The primary reviewer will then examine the abstracted data and organize it for group discussion based on the analytical framework within a preliminary report. This report will be discussed in person and subsequently revised.

1. For the purpose of this review, interventions are defined as anything that seeks to direct change in a directed manner. [↑](#footnote-ref-1)
2. These terms were chosen to reflect topics areas that were thought to be areas of greater research focus and thus topics that may yield further reviews, and were not topics currently being covered in other WHO research prioritization exercises (e.g. violence against women, female genital mutilation, and child marriage) [↑](#footnote-ref-2)
3. SRH outcomes of interest to those discussed in the WHO reproductive health strategy – family planning, unsafe abortion, STIs, HIV, RTIs, GBV, menstrual conditions, urinary and faecal incontinence due to obstetric fistula, uterine prolapse, pregnancy loss, sexual dysfunction, female genital mutilation, antenatal, perinatal, postpartum and newborn care, cervical cancer, infertility. [↑](#footnote-ref-3)
