# Supplementary material for: How Are Gender Equality and Human Rights Interventions Included in Sexual and Reproductive Health Programmes and Policies: A Systematic Review of Existing Research Foci and Gaps
Source: PLoS One. 2016 Dec 21;11(12):e0167542. doi: 10.1371/journal.pone.0167542 (PMC5176262; doi:10.1371/journal.pone.0167542)
Supplement: S1 Table — (DOCX) [file pone.0167542.s002.docx]

**Supporting Information Table 1. Gender-focused reviews**

| Citation | Type of review | Population | Geographic focus | Gender interventions covered | SRH topics | Outcomes assessed | Research Gaps/Limitations |
| --- | --- | --- | --- | --- | --- | --- | --- |
| Alexander KAC, C. L.;Deatrick, J. A.;Jemmott, L. S. Moving beyond safe sex to women-controlled safe sex: a concept analysis. J Adv Nurs 2012,68:1858-1869. | Literature review | Women | Global | Behavioral prevention of sexually related issues among women using women-controlled safe sex mechanisms (WSCC), includes development of new technology and increasing access to protective options | SH | Product attributes; side effects; safety profile; acceptability; efficacy; ability to covertly use; access to methods; demand for methods; use of methods; partner communication and trust; male influence in sexual decision-making; self-efficacy; relationship power dynamics; sexual pleasure | **Methodological:** None identified. **Content:** Need for better understanding of how WCSS is situated in a place of female passivity and lack of responsibility or control over sexual expressions, safety, and desire; greater understanding needed regarding how to move beyond just giving women the technology for empowerment. |
| Arango DJ, Morton M, Gennari F, Kiplesund S, Ellsberg M. Interventions to Prevent or Reduce Violence Against Women and Girls: A Systematic Review of Reviews. In. Washington, DC: World Bank; 2014. | Systematic review | Women and girls | Global | Prevention of violence against women | GBV | Access to services; incidence of child sexual abuse; children's self-protective behavior; knowledge and attitudes around child sexual assault; knowledge of protective behaviors; aggressive behavior; dependency; fearfulness towards strangers; bed-wetting; attitudes towards harmful traditional practices; intentions of mothers to have daughters undergo FGM; revictimization rates; VAW repeat perpetration rates; experience of IPV; pregnancy coercion; self-reported perpetration of violence; identification of survivors of IPV; knowledge and use of services; rape-related knowledge and attitudes; incidence of non-partner sexual assault; | **Methodological:** need for research evaluating longer-term effects; poor methodological rigor of studies, particularly among batterer intervention and sexual assault education programs; need for more consistent and reliable outcome measures (often self-report); no measurement of cost-effectiveness; no data on adaptation/replication of interventions. **Content:** less evidence from humanitarian and lower-income settings, particularly LAC, ME, and NA; limited documentation of unintended harm caused by interventions; limited research on child sexual abuse, human trafficking, or harmful traditional practices; limited focus on non-adult populations or indigenous or 'ethnically diverse' populations; fewer interventions target both men and women; no impact evaluations of economic empowerment or energy and water-related projects intending to reduce VAWG; less focus on primary prevention. |
| Barker G, Ricardo C, Nascimento M. Engaging men and boys in changing gender-based inequity in health: evidence from programme interventions. 2007. In. Geneva: World Health Organization; 2008. | Literature review | Men and boys | Global | Engaging men and boys in health | Fatherhood; GBV; HIV/AIDS; MNCH; SRH | Self-reported perpetration of violence; contraceptive use; communication with partner regarding child health, contraception, and RH decision-making; equitable treatment of children; use of SRH services; condom use; STI rates; social support of partner; exposure to messages/program; gender equitable attitudes | **Methodological:** heavy emphasis on pilot studies (i.e. lack of replication or scale-up); limited evaluation of long-term change; generally weak study designs; little data on cost; few GBV interventions evaluate (triangulate) change from female perspectives; lack of prioritization of indicator use (what are most linked to changes in gender relations?). **Content:** limited evidence on how male engagement approaches can be integrated with women's empowerment approaches and when is a combined approach more effective; limited understanding of policies or structural changes that could impact men and masculinity; Europe, Middle East, and North Africa least represented; few initiatives working with younger boys or with boys/men along their life course; limited evidence around fatherhood programs. |
| Barker G, Ricardo C. Young men and the construction of masculinity in sub-Saharan Africa: implications for HIV/AIDS, conflict, and violence. Washington, DC: World Bank; 2005. | Literature review | Men and boys | Botswana, Nigeria, South Africa, and Uganda | Programs applying a gender perspective to working with men/boys on HIV/AIDS and violence prevention | GBV; HIV/AIDS | Number of sexual partners; intention to use condoms; condom use; attitudes around safe sex; attitudes toward gender; self-reported STI symptoms; gender norms | **Methodological:** limited systematic documentation or evaluation of programs; often short-term programs/evaluations; limited rigor in evaluations; few large scale programs or examples of programs brought to scale. **Content:** fewer evaluations of GBV programs engaging men to address harmful gender norms. |
| Boender C, Santana D, Santillon D, Hardee K, Greene M, S S. the 'So What' report: A look at whether integrating a gender focus into programs makes a difference to outcomes. In. Washington, DC: IGWG; 2004. | Literature review | Women and men | Global | Integration of gender into reproductive health interventions | Abortion; FP; HIV/AIDS; STIs; MH | FP use; contraceptive knowledge; child mortality; fertility rate; teen pregnancy; age of marriage; use of skilled pregnancy care; maternal mortality; men's knowledge of wives' antenatal care; nutrition; STI incidence; quality of client-provider interaction; equitable gender attitudes; women's self-confidence; women's participation in leadership roles; women's decision-making power; women's mobility; women earning income; women's literacy; women's assertiveness in client-provider interactions; support from partners; partner communication about RH/FP | **Methodological:** limited rigor of evaluations; limited measurement of impact on gender; evaluations tend to be shorter-term than it takes to change gender norms. **Content:** few programs aim to improve gender equity (as opposed to just addressing gender); less evidence around topics other than STI/HIV prevention. |
| Cui RRL, R.;Thirumurthy, H.;Muessig, K. E.;Tucker, J. D. Microenterprise development interventions for sexual risk reduction: A systematic review. AIDS and Behavior 2013,17:2864-2877. | Literature review | Women | Global | Microenterprise development for gender equality | HIV/STIs | Prevalence or incidence of HIV/STIs; sexual risk behaviors (i.e. condom use, number of sex partners, etc.); household communication about HIV; economic well-being, self-empowerment; knowledge about physical and sexual violence | **Methodological:** generally weak study designs with short follow-up time periods; several studies only measured health related outcomes.  **Content:** lack of data on non-female populations; need to test interventions in more geographic settings (found studies in Haiti, India, Kenya, South Africa, US, and Zimbabwe). |
| Gerrits TS, M. Biomedical infertility care in sub-Saharan Africa: a social science-- review of current practices, experiences and view points. Facts Views Vis Obgyn 2010,2:194-207. | Systematic review | Women and men | sub-Saharan Africa | Male involvement to address gendered barriers to FP. Includes human rights lens that people have a right to infertility care. | FP (Infertility) | Quality of care; services offered; healthcare worker knowledge and attitudes; barriers to treatment seeking; cost of services; provider-patient communication; psychosocial impact of infertility; impact of gender roles on treatment seeking; acceptability of advanced reproductive technologies or donor material | **Methodological:** limited quantitative data (none on patients' experiences and needs); poor record keeping for outcomes. **Content:** limited focus on male infertility, including ethnographic studies on how to address cultural values that increase stigma towards men and/or reduce their treatment seeking behavior (e.g. pros and cons of male-only vs. couples counseling and resulting impact on existing gender inequities); implementation studies on how to introduce and improve introduction of advanced reproductive technologies in these settings in line with provision of these services as a human right (access, affordability, quality of care); need for studies on implementation of policy creation on infertility |
| Gill RS, D. E. Relevance of gender-sensitive policies and general health indicators to compare the status of South Asian women's health. Womens Health Issues 2011,21:12-18. | Literature review | Women | South Asia (Bangladesh, India, Nepal, Pakistan, and Sri Lanka) | Gender equitable policies related to health, education, economic, and political empowerment, and violence against women | GBV; FP; MH; RH | Maternal mortality ratio; antenatal care by trained CHWs; contraception rate; anemia rates; deliveries attended by skilled birth attendants; contraception rate; fertility rate; met need for family planning; health care decision-making; illiteracy rate; knowledge/impact of microfinance programs; HIV prevention knowledge; access to water; gender as % of budget; GDI rank and value; GEM rank and value; mean age at marriage; sex ratio at birth; seats in parliament held by women; mean age of marriage; experience of violence; female employment rate | **Methodological:** need for multisectoral approach, and thus multivariable analysis, to evaluate gender equity; inconsistent indicators to monitor gender equity and domestic violence; lack of indicators to assess health implications of policies on abortion or human trafficking; need to collect/compare baseline data to data on indicators post policy implementation to better assess success of gender-sensitive policies.  **Content:** need for evidence generation on implementation of policies to identify the reason they may not lead to improved gender equitable outcomes. |
| Guedes A. Addressing gender-based violence from the reproductive health/HIV sector: a literature review and analysis. In. Washington, DC: IGWG; 2004. | Literature review | Women, men, and adolescents | Low and middle-income countries | Programs addressing gender-based violence through behavior change communication, community mobilization, service provision, and policy. A segment of programs emphasize the need to view GBV as a human rights issue. | GBV | Awareness and attitudes towards GBV; gender norm attitudes; awareness of legislation; knowledge of services; communication about GBV; government discourse; implementation of legislation; mobilization of funds; knowledge, attitudes, and practices of providers related to IPV; confidentiality and privacy of services; quality of services; screening and detection rates; accuracy of service statistics; knowledge of policies; help seeking behaviors; sexual behavior; gender equity; prosecution of post conflict sexual assault; court decisions of sexual assault cases | **Methodological:** limited emphasis on measuring longer-term effects; limited rigorous evaluation design.  **Content:** few studies measuring impact of GBV initiatives on SRH outcomes; limited evaluation of GBV interventions in humanitarian settings. |
| Hanefeld J. How have Global Health Initiatives impacted on health equity? Promotion & education 2008,15:19-23. | Literature review | All | Global | Global Health Initiatives (i.e. PEPFAR, MAP, and GF) focused on HIV with gender equity components | HIV/AIDS | Women receiving ART, presence of gender in proposals and national frameworks, established gender equity targets, presence of gender disaggregated data | **Methodological:** Only anecdotal data on how programs address gender equity; limited consistency in collection of gender-focused or disaggregated data; need to understand longer-term impact of interventions on gender equity. **Content:** need for focus on understanding intervention impacts on social inequities, including negative consequences. |
| Integrating Multiple Gender Strategies to Improve HIV and AIDS Interventions: A Compendium of Programs in Africa. In. Washington, DC: International Center for Research on Women; 2009. | Literature review | Women and men | sub-Saharan African countries where PEPFAR operates | Gender-based programs to improve HIV services and mitigate men and women's vulnerability to HIV (e.g. reducing violence, addressing male norms, increasing women's legal protection, income and resources) | HIV/AIDS | Program reach; program participation; experience of IPV; gender equitable attitudes; access to resources; utilization of HIV services; HIV knowledge, attitudes, and behavior; contraceptive knowledge, attitudes, and practices; partner communication about HIV; sexual behavior; quality of care; client satisfaction | **Methodological:** limited rigor in data collection and evaluation; lack of measurement of gender indicators**. Content:** need for more research on increasing legal protection, on addressing male norms in programs other than GBV, and on evaluations of integrating gender into HIV treatment programs to improve access and adherence to treatment (e.g. impact of providing serves and times/locations convenient to men). |
| Jahan R. Restructuring the health system: experiences of advocates for gender equity in Bangladesh. Reproductive Health Matters 2003,11:183-191. | Literature review | Women | Bangladesh | Gender equitable policies, including shift from women's specific projects to gender mainstreaming approach | SRH | Female life expectancy, female infant mortality, maternal mortality, women receiving antenatal care, fertility rate, contraceptive prevalence | **Methodological:** need for better measures to understand aspects that will influence the implementation of gender equitable policies. **Content:** None identified. |
| Jennings LG, L. Influence of mhealth interventions on gender relations in developing countries: A systematic literature review. *International Journal for Equity in Health* 2013,12. | Systematic review | Women | Low and middle-income countries | mHealth interventions with a gender focus | Abortion; FP; HIV/AIDS; MH; SRH | Uptake of programs; women's social standing; HIV/AIDS related knowledge; hotline reach and participation; women's empowerment; women's status; women's resource control; mHealth intervention's feasibility, reach, and effect on contraception; couples' communication; male participation in women's SRH; effectiveness of mHealth platform's effectiveness to improve communication and patient follow-up; domestic violence; mobile stalking; privacy invasion; male partner distrust | **Methodological:** weak study designs (single group, post-test) limited study's ability to determine direct effects of the intervention. **Content:** gender relations only included in two study designs at outset; male perspective on gender relations not obtained; lack of reporting on change in gender relations over time; lack of data on whether changes in male engagement translated into more or less equitable outcomes for women. |
| Kennedy CEF, V. A.;O'Reilly, K. R.;Sweat, M. D. A systematic review of income generation interventions, including microfinance and vocational skills training, for HIV prevention. AIDS Care - Psychological and Socio-Medical Aspects of AIDS/HIV 2014,26:659-673. | Systematic review | All | Low and middle-income countries | Income generating interventions (some of which had a gender empowerment component) | HIV/AIDS | Condom use, HIV-related negotiation with partner, use of voluntary HIV testing and counseling services, HIV/STI knowledge, self-efficacy, accessing primary care for child health, partner communication, experience of IPV, number of sexual partners, attitudes toward sexual risk-taking behaviors, power in sexual relationships | **Methodological:** inconsistent use of rigorous study design; no measurement of HIV incidence or prevalence; lots of diversity in target populations, settings, study designs, and outcomes assessed, which limits ability to asses overall effectiveness programs; study follow-up length too short to assess outcomes. **Content:** limited focus on populations other than women; inconsistent focus on gender intervention components or outcomes - particular need to assess potential harms from empowerment; unknown effect of income generation on men/boys HIV outcomes. |
| Kraft JM, Wilkins KG, Morales GJ, Widyono M, Middlestadt SE. An evidence review of gender-integrated interventions in reproductive and maternal-child health. J Health Commun 2014,19 Suppl 1:122-141. | Systematic review | Women and men; couples | Low- and middle-income countries | Gender accommodating or transformative interventions addressing child survival (e.g. counseling to encourage male involvement; couples interventions to address communication or decision-making; women's educational or economic empowerment) | Child survival | School attendance by girls; reduced son preference; joint and equitable decision-making; men's support of ANC and PMTCT; partner communication; gender norms; fertility rates; uptake of and adherence to antenatal care interventions; family planning use; delivery at a health facility; skilled attendance at delivery; pregnancy care behaviors; maternal mortality; neonatal mortality; child stunting | **Methodological:** limited data on sustainability, replicability, and scale-up; limited data on which intervention components contribute to effects; lack of consistency around outcomes measured in male involvement programs (i.e. discussions with partners, joint decision-making, women's control over family resources, gender-role attitudes). **Content:** limited data on empowerment programs in sub-Saharan Africa; limited data on how community context or other conditions influence effects and outcomes; no GBV study measured MCH related outcomes; limited evidence on link between gender inequalities and child health outcomes. |
| Lee YMC, A.;Kocher, S. Factors related to risky sexual behaviors and effective STI/HIV and pregnancy intervention programs for African American adolescents. Public Health Nurs 2014,31:414-427. | Literature review | African American adolescents | United States | SRH education programs (some of which addressed gender roles) | ASRH | Violent behavior, school delinquency, drug use, recent sexual intercourse, condom use, SRH knowledge, multiple sexual partnerships, age of sexual debut | **Methodological:** No gender-related outcomes were measured. **Content:** limited understanding of how addressing gender roles contributed to findings. |
| Lee YMD, B.;Florez, E.;Holm, K. Factors related to sexual practices and successful sexually transmitted infection/HIV intervention programs for Latino adolescents. Public Health Nurs 2013,30:390-401. | Integrative literature review | Latino youth | United States, Mexico | Addressing traditional gender roles in sexual health | ASRH (not abortion) | Self-esteem; condom self-efficacy; decision-making; changes in risky sexual behavior (e.g. number of sexual partners, frequency of intercourse); condom use; STI rates; partner communication; age of onset of sexual activity; HIV testing; contraceptive use | **Methodological:** None identified. **Content:** Lack of information on how traditional Latino gender roles were addressed in these programs (e.g. was a transformative, accommodating, or harmful approach taken) and how they contributed to the study outcomes; need for better understanding of when to conduct multi-gender versus single gender interventions. |
| Mukherjee A, Das M. Mainstreaming gender in HIV programs: issues, challenges and way forward. EASTERN JOURNAL OF MEDICINE 2011,16:153-159. | Literature review | All | India | Gender mainstreaming within HIV interventions | HIV/AIDS | Level of incorporation of gender components; presence of gender sensitive indicators; presence of gender-disaggregated data; impact of incorporating GBV into HIV programming | **Methodological:** Limited baseline data on gender-focused initiatives; gender-sensitive indicators often not incorporated into program evaluation; due to other limitations, lack of ability to measure impact of gender mainstreaming on SRH outcomes. **Content:** limited integration of men as a focus of gender mainstreaming efforts. |
| Muralidharan A, Fehringer J, Pappa S, Rottach E, Das M, M M. Transforming Gender Norms, Roles, and Power Dynamics for Better Health: Evidence from a Systematic Review of Gender-integrated Health Programs in Low- and Middle-Income Countries. In. Washington, DC and Chapel Hill: Futures Group, Health Policy Project MEASURE Evaluation; 2014. | Systematic review | Women, men, and children | Low- and middle-income countries [focus on India] | Gender integrated programming to address RMNCH, HIV, GBV, universal health care | GBV; HIV/AIDS; Reproductive, maternal, neonatal, child and adolescent health | Age of marriage; age of sexual debut; attitudes towards GBV; knowledge, attitudes, and use of contraceptives; spousal communication about FP; sexual decision-making capacities; PMTCT; STI prevalence; HIV risk behaviors; knowledge of GBV and available services; breastfeeding; stunting; child immunization; gender equity; empowerment; masculinity; decision-making; vulnerability of girls; gender role attitudes | **Methodological:** limited evidence on scalability, particularly of gender-transformative programs; less emphasis on measuring changes in gender outcomes in accommodating programs; fewer RCTs in South Asia; less use of mixed methods in accommodating programs; higher proportion of transformative programs used a qualitative only design; few programs measured effects over a longer period of time.  **Content:** majority of programming implemented in sub-Saharan Africa and India and the majority of those in SSA focused on HIV; less attention to gender transformative approaches in safe motherhood, healthy timing and spacing of pregnancy, and neonatal, child health and nutrition; limited focus on the pathways or mechanisms through which gender impacted health |
| Phillips AFP, C. M. Moving beyond behaviour: Advancing HIV risk prevention epistemologies and interventions (a report on the state of the literature). Global Public Health 2011,6:577-592. | Literature review | Women and men | Global | Addressing issues of gender and HIV through masculinity and transactional sex (e.g. microcredit, awareness raising programs) | HIV/AIDS | Awareness of how ideas of masculinity can increase vulnerability to infection; IPV; access to ARVs | **Methodological:** None identified. **Content:** Need to evaluate programs addressing structural masculinity at regional and national levels; need clearer understanding of how addressing structural barriers to poverty and access to the marketplace among women can impact transactional sex; need for research on appropriate interventions in fragile states; need for research to understand how addressing structural capacity (e.g. healthcare infrastructure, governance, education system) can address structural barriers such as gender. |
| Ravindran T, Kelkar-Khambete A. Gender mainstreaming in health: looking back, looking forward. Global Public Health 2008,3:121-142. | Literature review | Women and men | Global | Gender mainstreaming in health (policies, programs, projects) and institutional mainstreaming (institutional goals, agenda setting, etc.). Gender mainstreaming framed as intending to address equity and equality between women and men based on the principle of health as a human right. | FP; GBV; HIV/STIs; MH; RH | Knowledge of RH services, condom use, contraceptive use, self-care, utilization of RH services, quality of services, gender relations, empowerment of women, awareness of gender issues | **Methodological:** limited comparison of baseline to post-intervention data; limited published data on the process of implementation or evaluation of gender mainstreaming interventions. **Content:** limited attention to dimensions of gender inequity in health such as morbidity, access to health care, and social and economic consequences of ill health. |
| Remme MS, M.;Vassall, A.;Heise, L.;Jacobi, J.;Ahumada, C.;Gay, J.;Watts, C. The cost and cost-effectiveness of gender-responsive interventions for HIV: A systematic review. Journal of the International AIDS Society 2014,17. | Systematic review | Women and men | Low and middle-income countries | HIV interventions with a gender focus (e.g. couples counselling, gender empowerment, cash transfers for schoolgirls, female condom distribution, PEP for rape survivors, etc.) | HIV/AIDS | Cost; cost-effectiveness; adherence; HIV incidence; condom use; unprotected sex; STI incidence; male reported problem drinking; male solicitation of transactional sex; male perpetration of IPV; HIV testing; reported STI symptoms; acceptance of violence; experience of physical or sexual violence; number of sexual partners; adolescent marriage rates; debut of sex | **Methodological: *i***mpact on biological outcomes not often measured; majority of cost data from single site or pilot studies **Content:** None identified. |
| Rottach E, Schuler S, K H. Gender Perspectives Improve Reproductive Health Outcomes: New Evidence. In. Washington, DC: IGWG; 2009. | Literature review | Women, men, and youth | Low- and middle-income countries | Integration of gender into reproductive health interventions | ASRH; FP; HIV/AIDS; STIs; harmful practices (e.g. FGM); MH | Contraceptive knowledge and use; fertility awareness; communication and joint decision-making; use of skilled pregnancy care; knowledge of HIV/AIDS; condom use; knowledge of STI symptoms; use of HIV/STI health services; knowledge, attitudes, and perpetration of IPV; knowledge, attitudes, and incidence of FGM; age of marriage; age of sexual debut; equitable gender attitudes and beliefs; women's self-confidence; women's participation; women's empowerment; women's decision-making power; girls education; women's mobility | **Methodological:** Growing, but still limited use of rigorous evaluation methodologies; need to measure change over longer spans of time to determine sustainability of results and need for follow-up activities; limited focus on measuring cost, replicability, or scalability of projects. **Content:** fewer evaluations in the Near East; difficulty isolating the effects of gender-equitable projects on RH/HIV/AIDS; heavy focus on measuring change in gender attitudes or women's empowerment; least amount of research in unintended pregnancies and maternal mortality; need for greater consideration of role of policies in reaching gender equality goals; need greater focus on gender transformative programs. |
| Shahmanesh M, Patel V, Mabey D, Cowan F. Effectiveness of interventions for the prevention of HIV and other sexually transmitted infections in female sex workers in resource poor setting: a systematic review. Tropical Medicine & International Health 2008,13:659-679. | Systematic review | Female sex workers | Low- and middle-income countries | Empowerment (for collective bargaining with police, brokers, brothel-owners); economic interventions; addressing gender disadvantages (female initiated methods of HIV/STI prevention) | HIV/AIDS, STIs | HIV incidence, STI incidence/prevalence, condom use, health service utilization | **Methodological:** limited rigor of studies (few RCTs, few with control groups); high study population attrition rates; limited data on exposure to intervention. **Content:** lack of evidence on the contribution of empowerment-based approaches to outcomes vs. policy; focus on only organized sex work, though much sex work occurs in unorganized settings. |
| Simbar M. Achievements of the Iranian family planning programmes 1956-2006. Eastern Mediterranean Health Journal 2012,18:279-286. | Literature review | Women | Iran | Activities designed to promote women's RH policies, such as enhancing women's participation in society, facilitating women's education and employment, promoting male participation in FP | FP | Total fertility rate; contraceptive prevalence rate; population growth rate; unwanted pregnancy rate | **Methodological:** does not report on any indicators of women's empowerment.  **Content:** limited data on the role of empowerment specific activities on SRH outcomes. |
| Skevington SMS, E. C.;Gillison, F. B. A systematic review to quantitatively evaluate 'stepping stones': A participatory community-based HIV/AIDS prevention intervention. AIDS and Behavior 2013,17:1025-1039. | Systematic review | Women and men | Low and middle-income countries | Stepping Stones' intervention, which targets gender roles and promotes equitable relationships through a participatory learning approach to reduce HIV | HIV/AIDS | Infection incidence, condom use, alcohol use, multiple partners, partner communication, gender inequity, stigma, knowledge of HIV | **Methodological:** limited evidence regarding impact on biomedical outcomes; need for longer evaluations (over 5 years); limited consistency across measures or use of reliable scales to better measure self-reported behaviors **Content:** need to understand program impact on other populations, such as older adults; requires additional measures to better understand why programs have a greater impact on men. |
| Sternberg P, Hubley J. Evaluating men's involvement as a strategy in sexual and reproductive health promotion. Health Promotion International 2004,19:389-396. | Literature review | Heterosexual men | Global | Male involvement in SRH (HIV/STI prevention, avoidance of unwanted pregnancy, women's RH, VAW, and promotion of responsible fatherhood) through peer education, workplace interventions, educational campaigns; cogitative behavioral skills training; community outreach) | Child care; GBV; HIV/AIDS; STIs | Condom use; HIV/STI incidence; unprotected sex; extramarital sex; sex with sex workers; antenatal care use; incidents of violence; participation in household chores; involvement with children | **Methodological:** None identified. **Content:** Limited evidence on impact on male involvement in women's RH (e.g. antenatal care) and in preventing pregnancy, including their own contraceptive use or role in facilitating their partners' use; lack of evidence on impact of male involvement on women's empowerment or on men resisting male social norms of dominance; limited evidence on the impact on men's lives of these interventions, including on their own gender identity |
| Underwood CH, Z.;Van Lith, L. M.;Lengwe Kunda, J. E.;Mallalieu, E. C. Role of community-level factors across the treatment cascade: a critical review. J Acquir Immune Defic Syndr 2014,66 Suppl 3:S311-318. | Literature review | Women and men | Low and middle-income countries | Addressing community level factors (CLFs), including gender norms to target each level of the HIV/AIDS treatment cascade (e.g. communication activities designed to encourage HIV testing and access to treatment and care) | HIV/AIDS | Service utilization, consistent condom use, provider attitudes, community level knowledge of HIV, adherence, access to health services, disclosure, retention, rates of survival | **Methodological:** need for more quantitative evidence; need to validate gender norm scales in order to compare results of interventions addressing CLFs. **Content:** Limited evaluations of interventions designed to effect CLFs, including efforts to address gender equity; limited exploration of the pathways between community factors and HIV outcomes. |
