# Supplementary material for: How Are Gender Equality and Human Rights Interventions Included in Sexual and Reproductive Health Programmes and Policies: A Systematic Review of Existing Research Foci and Gaps
Source: PLoS One. 2016 Dec 21;11(12):e0167542. doi: 10.1371/journal.pone.0167542 (PMC5176262; doi:10.1371/journal.pone.0167542)
Supplement: S2 Table — (DOCX) [file pone.0167542.s003.docx]

**Supporting Information Table 2. Human rights focused reviews**

| Citation | Type of review | Population | Geographic focus | Gender interventions covered | SRH topics | Outcomes assessed | Research Gaps/Limitations |
| --- | --- | --- | --- | --- | --- | --- | --- |
| Bustreo F, Hunt P, Gruskin S, Eide A, McGoey L, Rao S, Songane F, Tarantola D, Unnithan M, Yamin AE, van Bolhuis A, Ferguson L, Halliday E, Kuruvilla S, Popay J, Sander G. Women’s and children’s health: evidence of impact of human rights. In. Geneva: World Health Organization; 2013. | Literature review | Women and children | Nepal, Brazil, Malawi, Italy | Explicit application of human rights principles (e.g. availability, accessibility, acceptability, quality, participation, equality and non-discrimination, accountability) to women and children's health policies, programs, and interventions | MCH; SRH | Access to emergency contraceptive care; access to modern contraception; child mortality; vaccination coverage | **Methodological:** need for multi-disciplinary, multi-method approach to evaluating HR interventions; limited clarity about methods and tools needed to measure impact; no studies on cost-effectiveness of these approaches. **Content:** limited research on the impact of HR approaches on health, specifically suggests research on intervention studies explicitly considering participation within a framework of an HR approach and studies on how non-discrimination can be used as a framework to promote health equity; limited data on differential impacts across population groups; fewer studies in Africa or North America explicitly linking participation to HR. |
| Kerrigan D, Kennedy CE, Morgan-Thomas R, Reza-Paul S, Mwangi P, Win KT, et al. A community empowerment approach to the HIV response among sex workers: effectiveness, challenges, and considerations for implementation and scale-up. The Lancet 2015,385:172-185. | Systematic review | Sex workers | Low and middle-income countries | Community empowerment of sex workers to encourage collective ability to overcome barriers to their health and human rights | HIV/STIs | HIV infection, STI infection, condom use with clients, exposure to empowerment activities | **Methodological:** weak study designs (mainly cross-sectional studies); need for better aggregate measure of community empowerment; need for more qualitative/ethnographic data and data collected by sex workers (i.e. participatory research processes). **Content:** limited measurement of community empowerment as a social process (currently measured as exposure to certain activities, rather than process of gaining leadership, decision-making power, engagement with the state, etc.); limited geographical variation in study location (India, Brazil, Dominican Republic). |
| MacCarthy S, Rasanathan JJ, Ferguson L, Gruskin S. The pregnancy decisions of HIV-positive women: the state of knowledge and way forward. Reproductive health matters 2012,20:119-140. | Systematic review | HIV-positive women | Global | Pregnancy needs, rights, decisions, and desires of HIV positive women | SRH | Unmet need for contraception; unplanned pregnancies; availability of emergency contraception; accessibility of contraception; effectiveness of contraception in combination with ARVs; condom use; occurrence of coerced sterilization; fertility desire; success of assisted reproductive technologies; pregnancy rates; accessibility of assisted reproductive technology; HIV transmission; PMTCT; use of abortion services; acceptability of provider-initiated HIV testing; effectiveness of linkages between HIV testing and other HIV services; attitudes of health care providers; comfort with providers; confidentiality of services; HIV status disclosure; fear of HIV stigma; | **Methodological:** need for more participatory research methods involving HIV-positive women. **Content:** need to better understand how men can be involved in PMTCT; lack of knowledge on the needs of HIV positive women in relation to unsafe abortion; limited research on consequences of discriminatory laws on health outcomes; limited research among key populations (sex workers, IDUs, HIV serodiscordent couples, migrants, etc.) regarding SRH rights; limited data around coerced sterilization; need for more research on making assisted reproductive technologies available, accessible, and affordable to HIV-positive women in low and middle-income contexts; need for better understanding of how to change health workers discriminatory attitudes. |
| Rodríguez M, Harris S, Willson K, Hardee K. Voluntary family planning programs that respect protect and fulfill human rights: A systematic review of evidence. In. Washington, DC: Futures Group; 2013. | Literature review | Women and men | Global [US excluded] | Interventions aimed at promoting voluntary rights-based FP OR rights-based interventions that increase FP demand or access. While not the focus, includes programs aimed at changing gender norms and power imbalances | FP | Support for FP; knowledge and use of FP services; quality of services; client satisfaction; contraceptive prevalence rate; shared decision-making on contraceptive use; knowledge of STIs; abortion rate; unintended pregnancy; unmet need for FP; community empowerment; STI prevalence; condom use; partner communication around FP; gender equitable attitudes; girls' school enrollment; age of marriage | **Methodological:** process oriented nature of some HR approaches, such as community participation, make measurement of their impact on health outcomes difficult.  **Content:** no interventions specifically framed as rights-based FP interventions were identified and thus no measurement of explicit rights-based outcomes included; most FP studies at the service-level related to HR principles focus on quality of care; limited evaluations of policy-level interventions; limited focus on adults within community-level interventions. |
| Overs C, Hawkins K. Can rights stop the wrongs? Exploring the connections between framings of sex workers’ rights and sexual and reproductive health. BMC international health and human rights 2011,11:S6. | Literature review | Sex workers | Global | Sex workers' rights - government policy and law; international frameworks and conventions; declarations and advocacy documents | FP; GBV; STIs; SRH | N/A | **Methodological:** None identified. **Content:** limited attention to male or transgender, migrant, drug using, or older sex workers; need for greater understanding of how laws/policies are enforced and enacted in various settings; little data regarding the impact on SRH, including access to services, of laws/policies related to sex work. |
